# Lipid-Lowering and Antioxidative Activities of Aqueous Extracts of *Ocimum sanctum* L. Leaves in Rats Fed with a High-Cholesterol Diet

**DOI:** 10.1155/2011/962025

**Published:** 2011-09-21

**Authors:** Thamolwan Suanarunsawat, Watcharaporn Devakul Na Ayutthaya, Thanapat Songsak, Suwan Thirawarapan, Somlak Poungshompoo

**Affiliations:** ^1^Physiology Unit, Department of Medical Sciences, Faculty of Science, Rangsit University, Pathumthani 12000, Thailand; ^2^Pharmacology and Toxicology Unit, Department of Medical Sciences, Faculty of Science, Rangsit University, Pathumthani 12000, Thailand; ^3^Department of Pharmacognosy, Faculty of Pharmacy, Rangsit University, Pathumthani 12000, Thailand; ^4^Department of Physiology, Faculty of Pharmacy, Mahidol University, Bangkok 10400, Thailand; ^5^Department of Pathology, Faculty of Veterinary Science, Chulalongkorn University, Bangkok 10330, Thailand

## Abstract

The present study was conducted to investigate the lipid-lowering and antioxidative activities of *Ocimum sanctum* L. (OS) leaf extracts in liver and heart of rats fed with high-cholesterol (HC) diet for seven weeks. The results shows that OS suppressed the high levels of serum lipid profile and hepatic lipid content without significant effects on fecal lipid excretion. Fecal bile acids excretion was increased in HC rats treated with OS. The high serum levels of TBARS as well as AST, ALT, AP, LDH, CK-MB significantly decreased in HC rats treated with OS. OS suppressed the high level of TABARS and raised the low activities of GPx and CAT without any impact on SOD in the liver. As for the cardiac tissues, OS lowered the high level of TABARS, and raised the activities of GPx, CAT, and SOD. Histopathological results show that OS preserved the liver and myocardial tissues. It can be concluded that OS leaf extracts decreased hepatic and serum lipid profile, and provided the liver and cardiac tissues with protection from hypercholesterolemia. The lipid-lowering effect is probably due to the rise of bile acids synthesis using cholesterol as precursor, and antioxidative activity to protect liver from hypercholesterolemia.

## 1. Introduction

Cardiovascular diseases, particularly coronary heart disease (CAD), have become a growing problem, especially in developing countries. Hypercholesterolemia is widely known as a dominant risk factor for the development of cardiovascular diseases [1, 2]. It has been reported that oxidative stress induced by reactive oxygen species, plays an important role in the etiology of several diseases including atherosclerosis and CAD [3]. Hyperlipidemia has also been found to induce oxidative stress in various organs such as the liver, heart, and kidney [4, 5]. To lower high blood cholesterol, a number of lifestyle changes are recommended including smoking cessation, limiting alcohol consumption, increased physical activity and diet control. However, most people could not successfully control their blood cholesterol because of the modern life style. Medication is, therefore, considered their last choice. Although several synthetic drugs are available, they have been reported to have serious adverse effects, particularly liver damage [6]. Moreover, they lack several desirable properties such as efficacy and safety on long-term use, cost, and simplicity of administration. These factors do not fulfill conditions for patients' compliance. Therefore, attention is being directed to the medicines of herbal origin with hypolipidaemic activity. There are several kinds of medicinal plants that contain antioxidant and lipid-lowering effect. Among them, *Ocimum sanctum* L. (OS) is very promising as it is routinely used as cooking vegetable, and also in treatments of several diseases by local peoples in various countries. Also, it has been shown that OS leaves decrease serum lipid profile in diabetic rats and normal albino rabbits [7, 8]. Moreover, OS leaves provided liver protection against carcinogens and prevented isoproterenol-induced myocardial damage in rats [9, 10]. Though OS leaves have organ protective effects against various stress conditions, research studies that provide evidence of its lipidemic and antioxidative effects to protect the primary risk organs from hypercholesterolemia are still lacking. It has been reported that aqueous extracts of OS leaves expressed strong antioxidant activity both *in vivo* and *in vitro* studies [11, 12]. Therefore, the present study was conducted to investigate lipid-lowering and antioxidative activities in liver and heart of aqueous extracts of OS leaves in rats fed with a diet rich in cholesterol. 

## 2. Materials and Methods

### 2.1. Extraction of *Ocimum sanctum* L. Leaves

OS fresh leaves were obtained from the Institute of Thai Traditional Medicine, the Ministry of Public Health of Thailand. Fresh leaves of OS were washed in tap water and then cut into small pieces. The cut leaves were then cleaned and dried at room temperature. Dried leaves of OS were ground into fine powder and extracted by water. The aqueous extracts were then frozen and dried, and dark-brown powder was obtained. After the extraction process, the percent yield of OS extracts was 13.25 g from 100 g of dried OS leaf powder. The aqueous extracts were collected and stored at 4°C before determination of their phenolic content.

### 2.2. Determination of Total Phenolic Content in *Ocimum sanctum* L. Leaf Extracts

The total phenolic content of OS leaf extract was determined using the Folin-Ciocalteu method. 0.1 mL of 10 mg/mL (w/v) of OS extract was added to 1 mL of 7%  Na_2_CO_3_ solution and mixed thoroughly; 0.1 mL of Folin-Ciocalteu reagent was then added to the mixture. Distilled water was added to reach 2.5 mL, then the mixture was allowed to stand for 90 min with intermittent shaking. The absorbance was measured at 750 nm in a Benchmark plus microplate spectrophotometer (Bio-Rad Laboratories (UK) Ltd). The total phenolic content was determined from the standard gallic acid calibration curve, and it was expressed as mg of gallic acid equivalent/g of dry weight of the OS extracts.

### 2.3. Animal Preparation

Male Wistar rats weighing between 90–120 g were purchased from the Animal Center, Salaya Campus, Mahidol University, Thailand. The rats were cared for in accordance to the principles and guidelines of the Institutional Animal Ethics Committee of Rangsit University, which is under The National Council of Thailand for Laboratory Animal Care. The rats were housed in a 12 hr light-dark cycle room with controlled temperature at 25 ± 2°C and fed with standard rat food and tap water *ad libitum*. According to our preliminary study, serious liver and cardiac impairment were clearly observed in the rats that had serum cholesterol at least three times higher than the normal level. To obtain this high serum cholesterol level, 2.5 g% (w/w) cholesterol powder was added to the food (HC diet). The food composition is shown in Table 1.

Three groups of seven rats were established including, Group I: normal control rats that were fed with normal diet for seven weeks; Group II: rats fed with HC diet for seven weeks; Group III: rats fed with HC diet for seven weeks, and aqueous extracts of OS was daily administered by intragastric intubation during the last three weeks.

From our previous study, a supplementation of 2% (w/w) dried OS leaf powder in rat's diet for three weeks showed a lipid-lowering effect and partially protection of the liver in diabetic rats [7]. The percent yield of OS leaves extracted by water was 13.25 g% of OS dried leaf powder, and the average amount of dried OS leaf powder consumed by each rat was approximately 4.45 g/kg bw/day. Therefore, the daily dose of aqueous extract administered in this study was calculated based on these values and was approximately 590 mg/kg bw/day. During the last three weeks of the experiment, water was daily fed to groups I and II, whereas group III was daily fed with aqueous extracts of OS. To improve absorption, food was withheld for two hours before the OS extracts or water was administered. Body weight and food consumption were weekly recorded for seven weeks. 

During the last week of the experiment, rats' feces were collected for 3 consecutive days to determine fecal lipids and bile acids excretion. The rats were fasted overnight and anesthetized by intraperitoneal injection with zolitil (40 mg/kgbw) plus xylazine (3 mg/kgbw). Blood was collected from the abdominal vein to determine the serum lipid profile including total cholesterol (TC), triglyceride, low density lipoprotein cholesterol (LDL-C), and high density lipoprotein cholesterol (HDL-C). The atherogenic index (AI) was later calculated as the ratio of [TC-(HDL-C)]/(HDL-C). The liver and heart were also isolated, cleaned, and weighed. Liver and fecal lipids were extracted by modified method of Folch et al. [13]. Fecal bile acids including cholic acid and deoxycholic acid, the primary fecal bile acids in rat, were extracted and assayed by modified method described by Mosbach et al. [14] and Rizvi et al. [15].

### 2.4. Biochemical Evaluation of Liver and Cardiac Injury

Liver function was evaluated by assessing serum alanine aminotransferase (ALT), aspartate aminotransferase (AST), and alkaline phosphatase (AP) levels. Cardiac injury was also evaluated by measuring serum lactate dehydrogenase (LDH) and creatine kinase MB subunit (CK-MB) levels.

### 2.5. Determination of Serum Lipid Peroxide

At the end of the experiment, the rats were anesthetized; their blood was then collected from the abdominal vein to determine serum thiobarbituric acid reactive substances (TBARS) by the method previously described [16, 17]. Serum TBARS was expressed as *μ*mole of malondialdehyde (MDA)/L.

### 2.6. Determination of Lipid Peroxide and the Activity of Antioxidant Enzymes in both the Liver and Heart

At the end of the study, the rats were anesthetized and the jugular vein was cannulated to perfuse ice-cold normal saline to remove the red blood cells. Immediately after perfusion, both the liver and heart were isolated, cleaned and weighed. Both organs were kept at −80°C until further analysis was done.

#### 2.6.1. Determination of Tissue Lipid Peroxide Content

Lipid peroxides in both the liver and heart were assessed with thiobarbituric acid reactive substances (TBARS) as previously described [16]. TBARS was expressed in nmole of malondialdehyde (MDA)/mg protein using 1,1,3,3-tetraethoxy propane as standard. Tissue protein levels were determined with Lowry's method [18].

#### 2.6.2. Determination of the Activity of Tissue Antioxidant Enzymes

Antioxidant enzymes such as glutathione peroxidase (GPx), catalase (CAT), and superoxide dismutase (SOD) were determined in the liver and heart. Liver and cardiac tissue homogenates were prepared by homogenizing the tissues in a 0.1 M phosphate buffer pH 7.4. The homogenate was then centrifuged at 3,000 rpm, 4°C for 10 min. The supernatant was collected and centrifuged again at 7,800 g, 4°C for 30 min. The supernatant fraction was collected and further centrifuged at 136,000 g, 4°C for 60 min. The final supernatant was then analyzed for estimation of GPx, CAT, and SOD activities using the procedures described by Tapple [19], Luck [20], and Winterbourn et al. [21], respectively.

### 2.7. Evaluation of Liver, Cardiac, and Aortic Tissues Morphology

The liver, heart, and thoracic aorta were isolated, cleaned, dried, and then fixed in a buffer solution of 10% neutral buffered formalin. As for histopathological observations, longitudinal sections of the myocardial tissue and thoracic aorta were cut at the macroscopic lesions. As for the liver, sections were cut through the macroscopic lesions including capsules. The sections were further cut to 5 *μ*m thickness and were stained with haematoxylin and eosin (H&E) [22].

### 2.8. Biochemical Assay

The total serum levels of total cholesterol, triglyceride and HDL-C were assayed by using an enzymatic kit (Gesellschaft Für Biochemica und Diagnostica GmbH, Germany). LDL-C was calculated by using the equation: LDL-C = [TC-(HDL-C)]-(triglyceride/5). The serum levels of AST, ALT, LDH, and CK–MB were measured by using an enzymatic kit (Randox Laboratories, UK). Total cholesterol and triglyceride contents in the liver and feces were determined using enzymatic kit.

### 2.9. Statistical Analysis

All values were presented as means ± SEM. The results were analyzed by ANOVA. Duncan multiple rank test was performed to determine statistical significance among groups by using SPSS software version 11.5. Significant difference was accepted at *P* < 0.05.

## 3. Results

**Figure 1:**
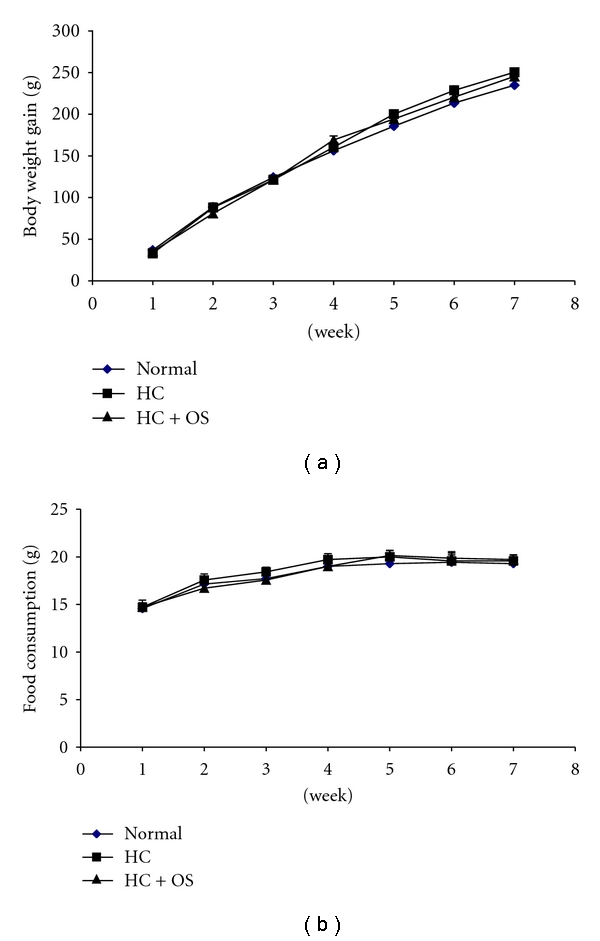
Gain of body weight and food consumption in all groups of rats.

The total phenolic content in aqueous extract of OS leaves was 90.4 ± 4.5 mg gallic acid equivalent/g of dried OS leaf extract.  Figure 1 shows changes of body weight gain and food intake throughout seven weeks. No significant differences of both body weight gain and food consumption were found among the groups of rats. Seven weeks of HC diet feeding raised liver weight without significant effect on heart weight (Table 1). The serum levels of TC, triglyceride, LDL-C, and AI were significantly increased, whereas HDL-C was significantly decreased in HC rats (Table 2). OS attenuated the high serum levels of TC, LDL-C, and AI and normalized triglyceride and HDL-C levels. HC diet significantly raised liver cholesterol and triglyceride content (Table 3). Fecal cholesterol, triglyceride, cholic acids, and deoxycholic acids excretion was significantly increased in HC rats as compared to normal rats. High level of liver lipid was lowered, whereas no significant change of fecal lipid excretion was obtained in HC rats treated with OS (Table 3). The high levels of both fecal cholic acids and deoxycholic acids were significantly decreased in HC rats treated with OS (Table 3).

The HC diet significantly raised serum ALT, AST, AP, LDH, and CK-MB (Table 4). As for HC rats treated with OS, high serum levels of ALT, AST, and AP were alleviated whereas CK-MB and LDH levels returned to normal levels. The high serum lipid peroxide as presented by TBARS was significantly decreased in HC rats treated with OS (Table 5). Liver TBARS was significantly increased, whereas GPx, CAT, and SOD activities were decreased in HC rats (Table 5). Treatment with OS significantly depressed the high level of liver TBARS in HC rats. In addition, OS increased the low levels of GPx and CAT activities to the normal level without affecting SOD activity. As for the cardiac tissue, TBARS was significantly increased while the activities of GPx and CAT were reduced in HC rats. The rats treated with OS showed a huge reduction in cardiac TBARS and normalized GPx and CAT activities. The cardiac SOD activity in HC rats treated with OS was markedly increased. 

**Figure 2:**
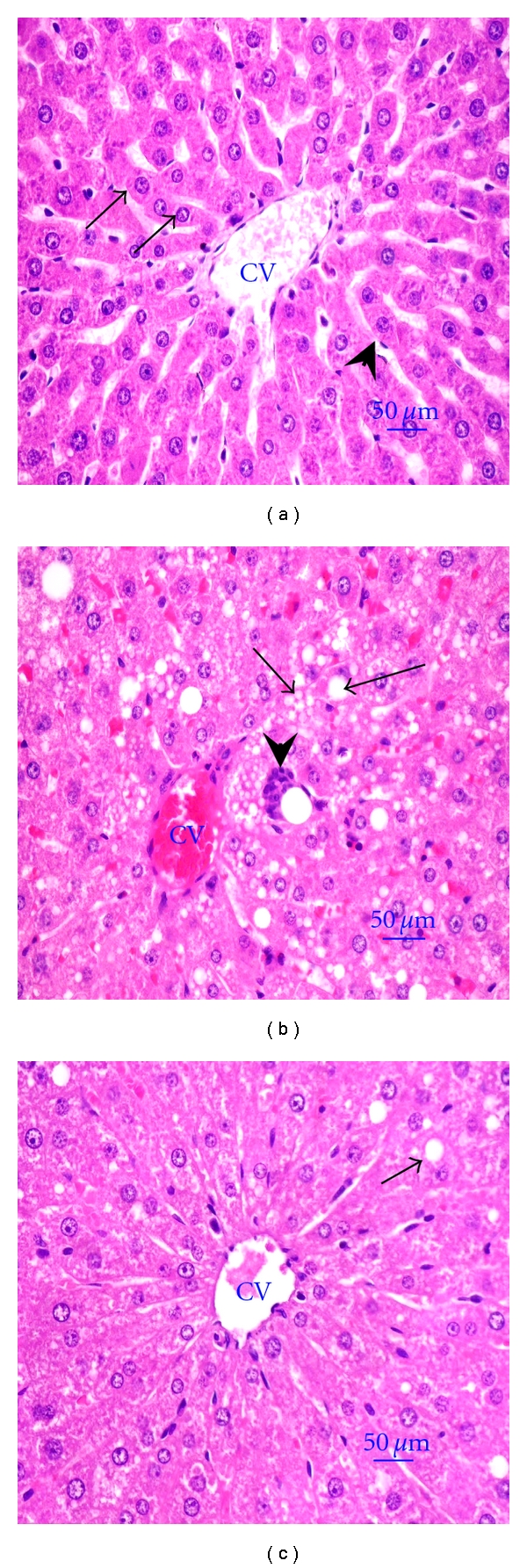
Histopathological appearance of liver (H&E×400). Normal hepatocyte had the round nucleus centrally (arrows); the flat endothelial cells (arrow-heads) are around the central vein (CV) (a). Diffuse vacuolar degeneration and necrosis of hepatocytes (arrows) and markedly focal fibrosis (arrow-head) were shown in HC rat (b). Hepatocytes of HC rat treated with aqueous extracts of OS leaves showed less injury of central vein and less fat vacuole (arrows) comparing to HC rat (c).

**Figure 3:**
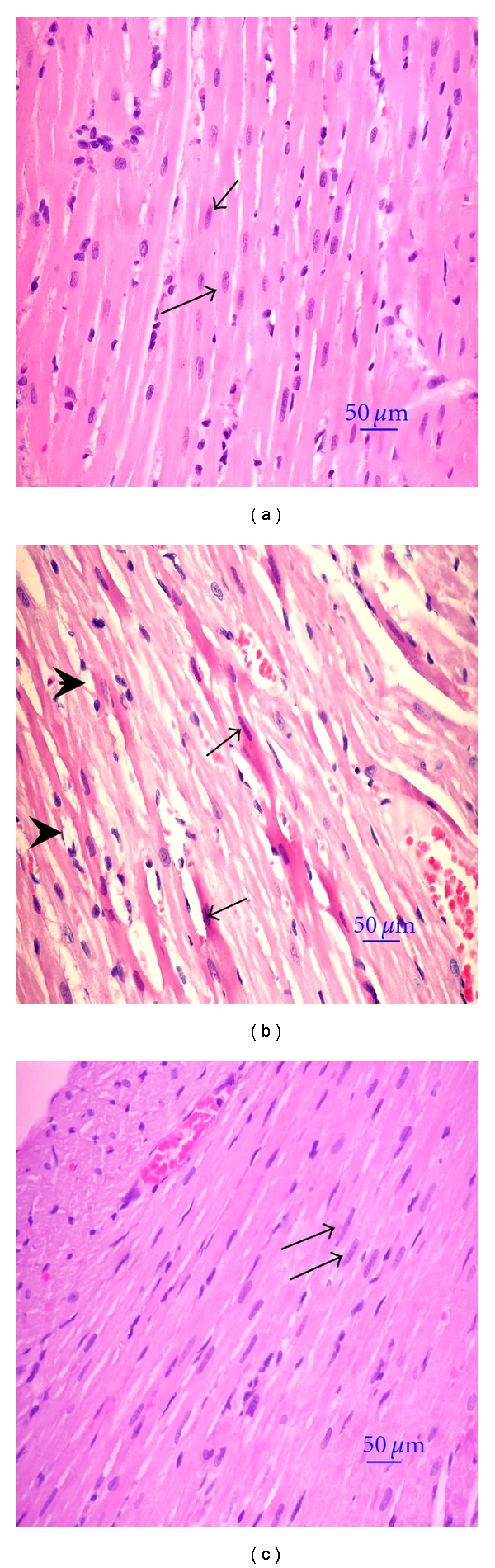
Histopathological appearance of myocardial cells (H&E×400). Oval-elongate nucleus centrally and homogeneous cytoplasm (arrows) in normal myocardial cell (a). Multi-focal vacuolar degeneration and necrosis of myocardial cells (arrow-heads) in HC rat (b). Apoptosis of myocardiocytes were also observed (arrow in b). Normal myocardial cell morphology with oval-elongate nucleus centrally and homogeneous cytoplasm (arrows) were shown in myocardiocytes of HC rats treated with aqueous extracts of OS leaves (c).

**Figure 4:**
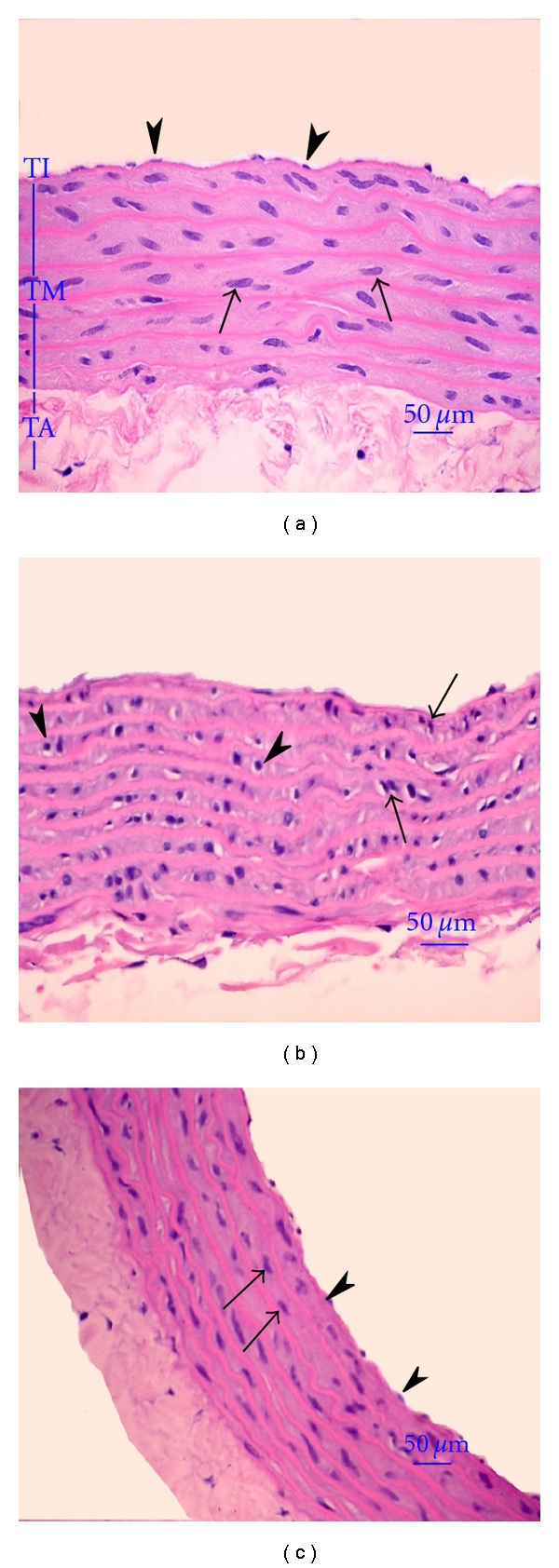
Histopathological appearance of thoracic aorta (H&E×400). Endotherial cells of normal rats were lining on the tunica intima (TI) (arrow-heads) (a). Most of the medial smooth muscle cells (arrows) of the tunica media (TM) oriented horizontal to the aortic canal. Normal amount of collagen fiber and connective tissue were exhibited in the tunica adventitia (TA). Multifocal degeneration, necrosis and disorientation of smooth muscle cells (arrows) in HC rats were shown (b). Multifocal mononuclear cell infiltration in the aortic wall (arrow -heads ) was also shown. No remarkable lesions of aortic tissue was observed in HC rats treated with aqueous extracts of OS leaves (c). Endothelial cells were lining on the tunica intima (arrow-heads). Most of the medial smooth muscle cells (arrows) of the tunica media oriented horizontal to the aortic canal. Normal amount of collagen fiber and connective tissue were exhibited in the tunica adventitia.

From histopathological analysis, the normal hepatocytes had centrally round nucleus and homogeneous cytoplasm (Figure 2(a)). There were flat endothelial cells around the central vein and sinusoid. Hepatocytes of HC rats showed severe degeneration with diffuse vacuolar degeneration and necrosis (Figure 2(b)). The endothelial lining of the central vein exhibited more cell injury with increased accumulation of fat vacuoles in the hepatocytes. Hepatic cells of HC rats treated with OS were improved with fewer endothelium injuries and less fat vacuoles (Figure 2(c)). Myocardiocyte of normal rats had oval-elongated nucleus centrally and homogeneous cytoplasm (Figure 3(a)). The HC rats exhibited a moderate dilation and thinning of the right ventricle wall with mild cardiac hypertrophy of the left ventricle. Multifocal vacuolar degeneration and necrosis were seen in the myocardial cells (Figure 3(b)). Apoptosis of myocardiocytes was also observed. In contrast, the myocardial cells of HC rats treated with OS reveal normal general appearance (Figure 3(c)). Normal amount of collagen fibers and connective tissues are exhibited in the tunica adventitia of the aortic tissue of normal rats (Figure 4(a)). Most of the medial smooth muscle cells of the tunica media oriented horizontally to the aortic canal. In contrast, multifocal degeneration, necrosis, and disorientation of smooth muscle cells were shown in the aortic tissue of HC rats (Figure 4(b)). No remarkable lesions were shown in aortic tissue of HC rats treated with OS extract (Figure 4(c)). 

## 4. Discussion

It has been widely known that elevation of serum cholesterol can lead to atherosclerosis; blood supply to the organs gradually diminishes until organ function becomes impaired. Several lines of evidence show that the improvement and incidence of atherosclerosis and CAD are associated with a lowering of serum cholesterol level [1, 23]. Although several interventions are recommended to treat hypercholesterolemia, people could not be successful because of the modern lifestyle. Therefore, medicines of herbal origin are interested by the investigators since they are enriched of bioactive phytochemicals that might be effective therapy, safe, and cheap. *Ocimum sanctum* L. (OS), commonly used as a cooking vegetable, has shown its potential as a therapeutic herb. The present study shows that seven weeks of HC diet feeding raised the serum lipid profile and AI. OS treatment attenuated the high serum lipid profile and AI. This implies that OS could be effective to alleviate atherosclerosis which then eventually prevents the occurrence of CAD. This is supported by the improvement of aortic tissue in HC rats treated with OS (Figure 2(c)). 

The liver is the primary organ responsible for maintaining cholesterol homeostasis. Several lines of evidence show that HC diet raises hepatic cholesterol content resulting in the increase of triglyceride synthesis [24, 25]. The hepatic lipid content and fecal lipid excretion as well as fecal bile acids excretion were evaluated in the present study to investigate the basic mechanism of hepatic lipid-lowering activity of OS. The reduction of hepatic lipid content and the elevation of fecal bile acid excretion without change of fecal lipid excretion in HC rats treated with OS indicate that hepatic lipid-lowering effect of OS was probably related to a lower intestinal bile acids absorption, resulting in an increase of hepatic bile acids biosynthesis using cholesterol as the precursor, and finally leading to the decrease of hepatic cholesterol and triglyceride accumulations. The reduction of hepatic lipid accumulation was supported by fewer fat vacuoles in the hepatocytes of HC rats treated with OS. Although OS decreased hepatic lipid content in HC rats, the liver weight did not reduce. The same result is shown by study of Yao et al. [26]. Other studies demonstrate that the liver weight was slightly reduced (12–20%), whereas hepatic cholesterol and triglyceride contents were lowered 26–50% and 31–37%, respectively [27, 28]. As shown in Table 2, the liver weight of HC rats was increased twice more than that of normal rats, whereas hepatic cholesterol and triglyceride contents increased 1,139% and 150%, respectively. OS decreased hepatic cholesterol and triglyceride content 25% and 16%, respectively, and might not affect the liver weight. From histopathological examination (Figure 4), the number of improved hepatocytes in HC rats treated with OS have increased and might be another cause of unchanged liver weight. 

Free radical-induced lipid peroxidation or oxidative stress has been shown to participate in the pathogenesis of several diseases [29, 30]. Hypercholesterolemia induces not only atherosclerosis but also produces a lot of free radicals in blood and tissues [30, 31]. TBARS level is a good indicator of lipid peroxidation. The present result shows that the serum TBARS was significantly elevated by HC diet administration, and this increase was attenuated by OS treatment, suggesting that OS decreases oxidative stress in HC rats. It is widely known that both the liver and heart are primary organs at risk from hypercholesterolemia. Retention of hepatic lipid has been shown to induce steatosis, and finally impairs hepatic function. Our results show that HC diet markedly suppressed hepatic and cardiac functions as expressed by an augmentation of serum levels of AST, ALT, AP, LDH, and CK-MB (Table 4). Hepatic and cardiac lipid peroxidation, as shown by the TBARS in both organs, also increased in HC rats. Moreover, antioxidant enzyme activities in both organs were depressed. These results reflect that seven weeks of an HC diet feeding induces oxidative stress to impair the liver and cardiac tissues. Recent findings indicate that some medical herbs have both a lipid-lowering ability and an antioxidative activities to suppress lipid peroxide production and then eventually may contribute to their effectiveness in preventing atherosclerosis and in protecting various organs at risk from hyperlipidemia [3, 5, 30]. OS was not only able to lower the serum and hepatic lipid but also to suppress the high serum levels of AST, ALT, LDH, and CK-MB. Moreover, lipid peroxidation was markedly suppressed, whereas the activities of antioxidant enzymes increased in both the liver and cardiac tissues of rats treated with OS. Our results indicate that OS had a free radical scavenging activity which probably provides organs protection from hypercholesterolemia. This is supported by histopathological examination of hepatocytes and myocardiocytes in rats treated with OS. The reduction of serum TBARS and liver, and increased hepatic antioxidant enzymes activities in rats treated with OS may be related to lower hepatic lipid accumulation. The present study clearly shows that OS may be of therapeutic importance, not only as a lipid-lowering agent in both serum and liver but also as a cytoprotective agent to protect the liver and cardiac injury from hypercholesterolemia. 

Interestingly, OS seems to normalize the cardiac tissue, whereas the liver tissue also improved but not to the normal appearance. It can be seen from the results that the serum levels of AST, ALT, and AP in HC rats were approximately 2–4 times higher than that of normal rats, whereas serum levels of LDH and CK-MB increased 1.3–1.5 times (Table 4). This reflects that the severity of liver impairment was more than that of the heart, and it might be possible to explain that OS improved but could not normalize the liver tissue in HC rats. Another interesting point is that OS normalized the activities of GPx and CAT in both the liver and cardiac tissues. However, the hepatic SOD activity remained low, whereas cardiac SOD activity markedly increased to higher than normal level in HC rats treated with OS. It can be seen from the results that HC diet feeding markedly decreased the hepatic SOD activity (61%), whereas the activities of hepatic GPx and CAT were slightly decreased (23–25%), indicating that hepatic SOD was so highly susceptible to steatosis, and this might be the reason why hepatic tissue improved but could not recover in HC rats treated with OS. Although OS decreased hepatic lipid content but the level might not be great enough to raise hepatic SOD activity. In contrast, the activity of cardiac SOD in untreated HC rats was slightly decreased and the level was not statistically significantly different from normal rats, hence it could be recovered by OS. The real mechanism why cardiac SOD activity in HC rats treated with OS was markedly higher than that of normal rats cannot yet be explained by the present study. It is known that SOD catalyses superoxide anions to hydrogen peroxide, which is broken down by CAT and GPx, and then prevents further generation of free radicals. Several studies show that HC diet raised cardiac superoxide anions without significant change of cardiac SOD activity [31, 32]. As shown in Table 5, HC diet feeding markedly raised TBARS level (83%), whereas it slightly decreased the activities of GPx, CAT, and SOD in cardiac tissue (16–35%). This means that HC induces a lot of free radicals production in cardiac tissue. OS should markedly enhance cardiac SOD activity in order to catalyze excess free radicals produced in cardiac tissue of HC rats. 

Several lines of evidence showed that plants with phenolic compounds had antilipidemic and antioxidative activities to protect the liver and heart against hyperlipidemia [33, 34]. The present study shows a significant amount of compounds with phenolic group in aqueous extracts of OS leaves that might be responsible for lipid-lowering and antioxidative actions to protect the liver and heart from hypercholesterolemia. However, it has not yet been known what kinds of phenolic compounds that participate in both actions; hence, they should be further identified.

In conclusion, aqueous extracts of OS leaves was able to decrease the high serum lipid profile and AI levels in rats fed with HC diet for seven weeks. OS decreased hepatic lipid content whereas increased fecal bile acids excretion without significant change of fecal lipid excretion. It also suppressed the high level of lipid peroxidation in the serum, liver, and cardiac tissues. OS significantly enhanced the activities of the antioxidant enzymes in both the liver and cardiac tissues. Our data indicate that the treatment of aqueous extracts of OS leaves during the last three weeks was powerful to attenuate both serum and hepatic lipid profile and provided the liver and cardiac protection from hypercholesterolemia. The basic mechanism of serum and hepatic lipid-lowering effect is probably due to the rise of bile acid synthesis using cholesterol and the antioxidative activity to protect liver from hypercholesterolemia. Phenolic compounds containing in aqueous extracts of OS leaves might be responsible for both lipid-lowering and antioxidative actions to protect the liver and heart from hypercholesterolemia. 

## Figures and Tables

**Table 1:** Dietary composition of normal and high-cholesterol diet.

Food ingredients	Normal diet	HC diet
	(g/100 g diet)	(g/100 g diet)
Crude protein	27	27
Soy bean oil	4.5	4.5
Fiber	5	5
Corn starch	58.5	49.5
Mineral mix	4.3	4.3
Vitamin mix	0.34	0.34
Choline chloride	0.15	0.15
Cholesterol powder	—	2.5
Cholic acid	—	0.6
Palm oil (mL)	—	6

**Table 2:** Changes of liver weight, heart weight, and serum lipid profile in normal rats, HC rats, and HC rats treated with aqueous extracts of *Ocimum sanctum* L. leaves (OS).

	Group		
	Normal	HC	HC + OS
Organ weight (g/kgbw)			
Liver	32.5 ± 0.6^a^	62.2 ± 1.5^b^	63.1 ± 1.7^b^
Heart	3.53 ± 0.1^a^	3.33 ± 0.09^a^	3.46 ± 0.12^a^
Serum lipid (mg/dL)			
Total cholesterol	38 ± 2^a^	133 ± 5^b^	96 ± 9^c^
Triglyceride	25 ± 1^a^	50 ± 7^b^	26 ± 4^a^
HDL-C	20 ± 0^a^	17 ± 1^b^	20 ± 1^a^
LDL-C	13 ± 1^a^	105 ± 6^b^	71 ± 9^c^

AI	0.9 ± 0.1^a^	6.9 ± 0.8^b^	4.0 ± 0.6^c^

Values are shown as means ± SEM of seven rats per group.

Values with different superscripts in the same row are significantly different at *P* < 0.05. HC: high cholesterol.

**Table 3:** Changes of liver and fecal lipid excretion, and fecal bile acids excretion in normal rats, HC rats, and HC rats treated with aqueous extracts of *Ocimum sanctum* L. leaves (OS).

	Group		
	Normal	HC	HC + OS
Liver lipids content (mg/g liver)			
Total cholesterol	2.8 ± 0.1^a^	34.7 ± 2.6^b^	26.1 ± 0.9^c^
Triglyceride	8.25 ± 0.40^a^	20.6 ± 0.8^b^	17.2 ± 1.5^c^

Fecal lipids excretion (mg/g feces)			
Total cholesterol	3.6 ± 0.2^a^	48.0 ± 2.3^b^	44.6 ± 3.4^b^
Triglyceride	1.5 ± 0.1^a^	2.8 ± 0.3^b^	2.6 ± 0.3^b^

Fecal bile acids excretion (mg/g feces)			
Cholic acid	0.22 ± 0.03^a^	0.56 ± 0.06^b^	0.89 ± 0.05^c^
Deoxycholic acid	0.17 ± 0.02^a^	0.50 ± 0.03^b^	0.84 ± 0.04^c^

Values are shown as means ± SEM of seven rats per group.

Values with different superscripts in the same row are significantly different at *P* < 0.05. HC: high cholesterol.

**Table 4:** Changes of serum alanine aminotransferase (ALT), aspartate aminotransferase (AST), alkaline phosphatase (AP), lactate dehydrogenase (LDH), and creatine kinase MB subunit (CK-MB) in normal rats, HC rats, and HC rats treated with aqueous extracts of *Ocimum sanctum* L. leaves (OS).

	Group		
	Normal	HC	HC + OS
ALT (U/L)	25 ± 1^a^	118 ± 17^b^	61 ± 8^c^
AST (U/L)	67 ± 3^a^	166 ± 14^b^	103 ± 5^c^
AP (U/L)	116 ± 8^a^	207 ± 6^b^	182 ± 9^c^
LDH (U/L)	530 ± 78^a^	825 ± 86^b^	380 ± 62^a^
CK-MB (U/L)	499 ± 56^a^	685 ± 35^b^	421 ± 39^a^

Values are shown as means ± SEM of seven rats per group.

Values with different superscripts in the same row are significantly different at *P* < 0.05. HC: high cholesterol.

**Table 5:** Effect of aqueous extracts of *Ocimum sanctum* L. leaves on serum lipid peroxide, hepatic and cardiac tissue lipid peroxide and antioxidant enzymes activity in HC rats.

	Group		
	Normal	HC	HC + OS
Serum TBAR (*μ*mole/L)	1.8 ± 0.08^a^	2.7 ± 0.04^b^	2.1 ± 0.1^c^
Liver			
TBARS (nmoleMDA/mg protein)	0.88 ± 0.06^a^	1.38 ± 0.04^b^	0.59 ± 0.05^c^
GPx (*μ*mole/min/mg protein)	1.3 ± 0.06^a^	0.97 ± 0.02^b^	1.19 ± 0.03^a^
CAT (*μ*mole/min/mg protein)	360 ± 16.6^a^	275 ± 18^b^	340 ± 28^a^
SOD (unit/mg protein)	103 ± 13.9^a^	40.1 ± 3.29^b^	54.4 ± 2.32^b^
Heart			
TBARS (nmoleMDA/mg protein)	1.0 ± 0.05^a^	1.83 ± 0.05^b^	0.61 ± 0.07^c^
GPx (*μ*mole/mg protein)	0.28 ± 0.02^a^	0.20 ± 0.02^b^	0.30 ± 0.02^a^
CAT (*μ*mole/min/mg protein)	6.95 ± 0.38^a^	5.82 ± 0.31^b^	7.33 ± 0.47^a^
SOD (unit/mg protein)	25.3 ± 2.1^a^	14.3 ± 1.2^a^	82.8 ± 11.7^c^

Values are shown as means ± SEM of seven rats per group.

Values with different superscripts in the same row are significantly different at *P* < 0.05.

TBARS: thiobarbituric acid reactive substances; GPx: glutathione peroxidase; CAT: catalase; SOD: superoxide dismutase.

## Abbreviations

HC: High cholesterol
OS: *Ocimum sanctum* L.
AI: Atherogenic index
ALT: Alanine aminotransferase
AST: Aspartate aminotransferase
AP: Alkaline phosphatise
LDH: Lactate dehydrogenase
CK-MB: Creatine kinase MB subunit
TBARS: Thiobarbituric acid reactive substances
GPx: Glutathione peroxidise
CAT: Catalase
SOD: Superoxide dismutase.
